# Evolution of the Antiretroviral Restriction Factor TRIMCyp in Old World Primates

**DOI:** 10.1371/journal.pone.0014019

**Published:** 2010-11-16

**Authors:** Elizabeth A. Dietrich, Lisa Jones-Engel, Shiu-Lok Hu

**Affiliations:** 1 Department of Microbiology, University of Washington, Seattle, Washington, United States of America; 2 Washington National Primate Research Center, University of Washington, Seattle, Washington, United States of America; 3 Department of Pharmaceutics, University of Washington, Seattle, Washington, United States of America; University of Pittsburgh, United States of America

## Abstract

The retroviral restriction factor TRIMCyp, which is a fusion protein derived from the *TRIM5* gene, blocks replication at a post-entry step. Among Old World primates, TRIMCyp has been found in four species of Asian macaques, but not in African monkeys. To further define the evolutionary origin of Old World TRIMCyp, we examined two species of baboons (genus *Papio*) and three additional macaque species, including *M. sylvanus*, which is the only macaque species found outside Asia, and represents the earliest diverging branch of the macaque lineage. None of four *P. cynocephalus anubis*, one *P. hamadryas*, and 36 *M. sylvanus* had either TRIMCyp mRNA or the genetic features required for its expression. *M. sylvanus* genomic sequences indicated that the lack of TRIMCyp in this species was not due to genetic homogeneity among specimens studied and revealed the existence of four TRIM5α alleles, all distinct from *M. mulatta* and *Papio* counterparts. Together with existing data on macaque evolution, our findings indicate that TRIMCyp evolved in the ancestors of Asian macaques approximately 5–6 million years before present (ybp), likely as a result of a retroviral threat. TRIMCyp then became fixed in the *M. nemestrina* lineage after it diverged from *M. nigra*, approximately 2 million ybp. The macaque lineage is unique among primates studied so far due to the presence and diversity of both TRIM5 and TRIMCyp restriction factors. Studies of these antiviral proteins may provide valuable information about natural antiviral mechanisms, and give further insight into the factors that shaped the evolution of macaque species.

## Introduction

Primates have been infected with retroviruses frequently throughout their evolution. Retroviral infections are believed to have driven the evolution of host factors such as the restriction factors TRIM5α and TRIMCyp [1]. These restriction factors specifically inhibit retroviral replication [2]–[5], and bear the marks of previous evolutionary conflicts [6], [7].

TRIM5α and TRIMCyp are two of several alternatively spliced isoforms of the *TRIM5* gene [8]. This gene belongs to the tripartite motif (*TRIM*) gene family, of which several members in addition to *TRIM5* have been implicated in immune responses to pathogens [9]. TRIM proteins contain, in order, a RING domain, one or two B-Box domains, and a coiled coil domain. TRIM5α also has a C-terminal B30.2/SPRY domain, which recognizes and binds to the capsids of susceptible retroviruses, leading to post-entry restriction of infection [8], [10], [11]. This restriction occurs in a two-stage process, with stages both before and after reverse transcription [2], [4], [12].

In a striking instance of convergent evolution, cyclophilin A (*CypA*) sequences have been inserted into the *TRIM5* gene by independent retrotransposition events in both New World (*Aotus*/owl monkey) and Old World (*Macaca*/macaque) primate lineages. Alternative splicing to these sequences leads to the production of TRIMCyp, in which the B30.2/SPRY domain of TRIM5α is replaced with a CypA domain. Because CypA, like the B30.2/SPRY domain, can bind to retroviral capsids, TRIMCyp also has antiretroviral activity [13], [14]. New World and Old World TRIMCyp proteins have distinct antiretroviral specificities, which also differ from that of TRIM5α [15]–[18].

**Figure 1:**
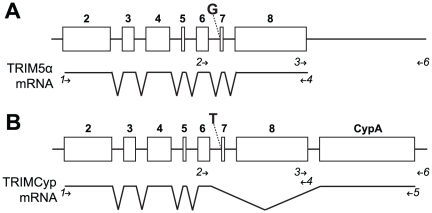
Genomic organization and mRNA splicing of *TRIM5* alleles. (A) DNA sequence of the TRIM5α-expressing allele is shown schematically on top, with open boxes representing exons 2–8 numbered in bold. The mRNA splicing pattern indicated below. The canonical splice acceptor sequence with a G nucleotide upstream of exon 7 is as indicated. (B) The *CypA* insertion in the TRIMCyp-expressing allele is located as indicated. A SNP (T) at the exon 7 splice acceptor allows the splicing of exon 6 to the coding region of the *CypA* insertion. Minor splice isoforms in both alleles are not depicted. Primers used for analyses described in subsequent figures and text are depicted as arrows and numbered in italics. Primers used for genomic analysis are shown above the mRNA diagram, and those for RT-PCR analysis shown below. Primer 4 was used for both analyses.

In macaques, the retrotransposed *CypA* sequence required for TRIMCyp production is found in the 3′ untranslated region (UTR) of the *TRIM5* gene. TRIMCyp expression and the presence of this *CypA* insertion are correlated with a single nucleotide polymorphism (SNP) at the exon 7 splice acceptor site, in which the canonical AG dinucleotide splice acceptor is changed to AT [19]. This change leads to the production of alternatively spliced products including TRIMCyp, which results from skipping of exons 7 and 8 and splicing to the *CypA* insertion (Figure 1) [15]–[19].

TRIMCyp, and the splice acceptor SNP and *CypA* insertion required for its expression, have been found in all four species of Asian macaques that have been tested so far. In these four species, *Macaca nemestrina*, *M. leonina*, *M. mulatta*, and *M. fascicularis*, the genetic changes are found at varying frequencies. They appear to be fixed in *M. nemestrina* and the closely related *M. leonina* [16], [19]–[21]. In *M. mulatta*, TRIMCyp is present in animals of Indian origin at an allele frequency of approximately 25%. It has not been found in Chinese *M. mulatta*, among at least 76 individuals that have been screened [18], [20].

Phylogenetically, *M. mulatta* and *M. fascicularis* belong to the *fascicularis* group of Asian macaques. *M. nemestrina* and *M. leonina* belong to the earliest diverging group, the *silenus* group [22], [ 23; See Figure 6]. Thus, TRIMCyp is present in maximally divergent groups within the Asian macaques, and was likely present in the ancestor of all Asian macaques. TRIMCyp was not found in sooty mangabeys (*Cercocebus atys*), the only other Old World monkey species that has been tested [20]. This African monkey species, along with baboons (genus *Papio*), belongs to the papionin clade, a sister clade to the macaques. However, the absence of TRIMCyp in sooty mangabeys does not necessarily imply that it is absent in the papionin clade as a whole, because at least one Asian macaque lineage (Chinese *M. mulatta*) also appears to lack TRIMCyp. Therefore, studies of additional Old World primate species are necessary to help establish the evolutionary origin of TRIMCyp.

*M. sylvanus*, the only African macaque, has a unique position within the macaque lineage. This species diverged from the Asian macaques after the macaques diverged from the papionin clade, making it the most closely related outgroup to the Asian macaques. Thus, study of *M. sylvanus* will help to determine whether TRIMCyp evolved before or after the divergence of African from Asian macaques.

In this study, we tested *M. sylvanus*, two baboon species, and two additional Asian macaques, *M. nigra* and *M. thibetana*, for TRIMCyp. We find that all samples lack the *CypA* insertion in the *TRIM5* 3′ UTR, the splice site SNP associated with TRIMCyp, and TRIMCyp expression. These findings indicate that TRIMCyp likely evolved in Old World primates after the divergence of *M. sylvanus* from the Asian macaques, approximately 5–6 million years before present (ybp). It then became fixed in *M. nemestrina* and *M. leonina* after their divergence from *M. nigra*, approximately 2 million ybp. Identification of the evolutionary origin of TRIMCyp in Old World primates suggests that retroviral selection may have helped to shape the speciation of Asian macaques.

## Results

### Baboons lack TRIMCyp and the *CypA* insertion

Old World primate TRIMCyp has so far been found in Asian macaques, and not in sooty mangabeys, which are African primates that belong to the papionin clade, a sister clade to the macaques. Because the frequency of the TRIMCyp allele is variable among Asian macaques, we reasoned that it may also be present in species related to sooty mangabeys. To test this possibility, we examined baboons, which also belong to the papionin clade. We tested five baboons, including four *P. cynocephalus anubis* and one *P. hamadryas*, for the *TRIM5* exon 7 splice site SNP, which is required for TRIMCyp expression. As controls, we used three *M. fascicularis* animals of known genotype. We initially used the restriction assay developed by Newman *et al.* [20], which takes advantage of a second polymorphism upstream of the splice site. In macaques, the presence of an upstream *NsiI* restriction site is linked to the T allele at the splice site, and thus correlated with TRIMCyp expression. The absence of the *NsiI* site is linked to the G allele and correlated with the absence of TRIMCyp.

**Figure 2:**
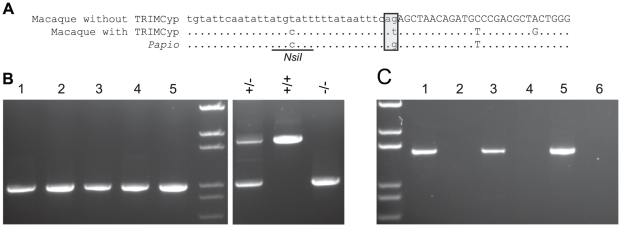
Baboons lack TRIMCyp. (A) Sequence of the intron 6/exon 7 junction, showing the splice site SNP and the *NsiI* polymorphism, in a macaque that expresses TRIMCyp (*M. nemestrina*, GenBank EU371641.1); a macaque lacking TRIMCyp (*M. mulatta* sequenced genome, GenBank NC_007871.1); and baboons (*Papio*, GenBank HM468444-HM468446). Capital letters, exon; lowercase letters; intron. Box, splice acceptor site. DNA from both *P. cynocephalus anubis* and *P. hamadryas* was sequenced, and all sequences were identical in the region shown here. (B) PCR across the *TRIM5* 3′ UTR (Primers 3 and 6) in *P. cynocephalus anubis* (4 individuals, lanes 1–4) and *P. hamadryas* (lane 5). Three *M. fascicularis* of known genotypes were used as controls. Lane 6, *CypA* insertion heterozygote. Lane 7, homozygote with *CypA* insertion. Lane 8, homozygote lacking *CypA* insertion. (C) RT-PCR for TRIM5α (lanes 1, 3, and 5) and TRIMCyp (lanes 2, 4, and 6) in cDNA from 3 *P. cynocephalus anubis*.

However, we found that this correlation does not apply in baboons. All five *Papio* samples had the *NsiI* restriction site at the expected location, but sequencing of this region of the genome demonstrated that these animals have the G allele at the splice site (Figure 2a) (GenBank HM468444-HM468446). Therefore, while the restriction assay is useful for genotyping macaques, it is not valid for baboons and should be verified when testing any new species.

We tested the same animals for the *CypA* insertion, using PCR primers designed to bind on either side of the putative *CypA* sequence in the 3′ UTR of the *TRIM5* gene (Primers 3 and 6). All five baboons had only the shorter PCR product, demonstrating the absence of the *CypA* insertion (Figure 2b). *P. cynocephalus anubis* individuals for which RNA was available also lacked TRIMCyp mRNA expression (Figure 2c). Thus, baboons, like sooty mangabeys, lack TRIMCyp expression and the *CypA* insertion. These data support the view that TRIMCyp is not present in the papionin clade, and that it evolved in the macaque lineage after its divergence from papionins.

### TRIMCyp evolved after the divergence of Asian macaques and *M. sylvanus*

**Figure 3:**
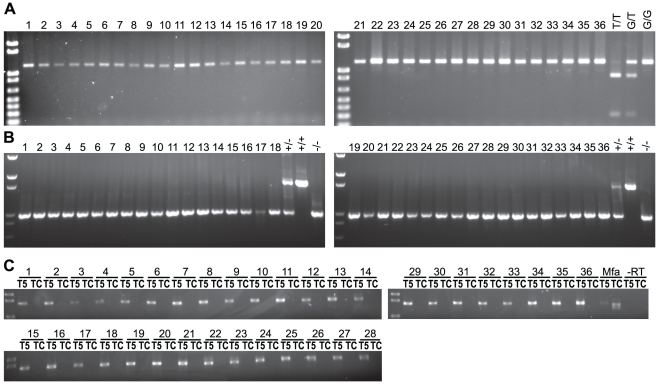
*M. sylvanus* lack TRIMCyp. (A) *NsiI* restriction site assay on 36 *M. sylvanus*. (B) PCR across the *TRIM5* 3′ UTR in 36 *M. sylvanus*. Three *M. fascicularis* of known genotype were used as controls (last 3 lanes of both parts A and B). (C) RT-PCR for TRIM5 (left lanes) and TRIMCyp (right lanes) in cDNA from 36 *M. sylvanus*. Mfa, *M. fascicularis* heterozygote expressing both TRIM5 and TRIMCyp. –RT, *M. sylvanus* sample run without reverse transcriptase.

In order to further define the evolutionary origin of TRIMCyp among Old World primates, we examined *M. sylvanus*, which represents the earliest diverging macaque species. We sampled 36 *M. sylvanus* individuals from Gibraltar [24]. Unlike the baboons, all *M. sylvanus* lacked the *NsiI* restriction site (Figure 3a). Sequence analysis confirmed that they had the G allele at the splice site. Thus, the genetic linkage between the *NsiI* site and the SNP associated with TRIMCyp expression appears to be conserved among macaque species, including *M. sylvanus*, but not in baboons. As expected from these results, none of the *M. sylvanus* tested had either the *CypA* insertion (Figure 3b) or TRIMCyp mRNA expression (Figure 3c). We sequenced the PCR products shown in Figure 3b for eight animals. These sequences were similar to *M. mulatta* sequences known to lack TRIMCyp, and showed no evidence of any deletions or rearrangements in this region (data not shown). Thus, it is unlikely that the *CypA* insertion was present in these animals but deleted at the sequence level. This suggests that TRIMCyp evolved after the divergence of *M. sylvanus* from the Asian macaques.

### *M. sylvanus* TRIM5α is polymorphic and distinct from orthologues in closely related species

In order to determine the level of diversity among the *M. sylvanus* samples tested and to ensure that the sample population was not homogeneous, we cloned and sequenced TRIM5α cDNA from seven *M. sylvanus* (GenBank HM468429-HM468432). We found six SNPs, of which four result in amino acid substitutions and two are synonymous (Table 1). We also cloned and sequenced *TRIM5* exon 8 from genomic DNA in nine animals, including the same seven from which TRIM5α was cloned. At least four of nine animals were heterozygotes. Four of the SNPs (three nonsynonymous and one synonymous) are in the B30.2/SPRY domain, which is the capsid-binding domain and has been described as the most variable domain in other primate TRIM5α sequences [6], [7], [25]. The SNP at amino acid 339 (nucleotide 1016) is within the “patch” of amino acids previously described as being under strong positive selection in primate lineages [6]. The other two polymorphisms are in the RING and coiled-coil domains, respectively.

**Table 1:** Intraspecies polymorphisms in *M. sylvanus* TRIM5α.

SNP	1		2		3		4		5		6	
	nt	aa	nt	aa	nt	aa	nt	aa	nt	aa	nt	aa
Residue	90	30	**424**	**142**	**1016**	**339**	1071	357	**1072**	**358**	**1268**	**423**
Allele 1	T	C	**A**	**M**	**T**	**M**	C	S	**A**	**I**	**A**	**H**
Allele 2	C	C	**G**	**V**	**C**	**T**	T	S	**C**	**L**	**A**	**H**
Allele 3	C	C	**A**	**M**	**C**	**T**	T	S	**C**	**L**	**G**	**R**
Allele 4	T	C	**A**	**M**	**C**	**T**	T	S	**C**	**L**	**G**	**R**
Domain	RING				Coiled Coil				B30.2/SPRY			

Nonsynonymous SNPs are marked in **bold**.

nt  =  nucleotide residue number.

aa  =  amino acid residue number.

We also compared the predicted *M. sylvanus* TRIM5α amino acid sequence with all six common alleles identified in *M. mulatta* [7], and with two *P. cynocephalus anubis* TRIM5α sequences (Table 2). One of these baboon sequences was characterized for this study (GenBank HM468433), while the other was previously published [26]. We found 13 *M. sylvanus*-specific residues, suggesting that extensive evolution has occurred in this species since its divergence from a common ancestor. Two residues (P29 and E247) were shared with baboons but not with *M. mulatta*, and four (K44, A296, M330, and T339 in some alleles) were shared with *M. mulatta* but not baboons. Six residues (M142, M310, M339, L358, L385, and R423) were not found in any other available TRIM5α sequence, including those of apes and New World monkeys. Like the intraspecies polymorphisms, these interspecies differences were distributed throughout the length of the TRIM5α gene, with a large number found in the B30.2/SPRY domain. We also found intraspecies polymorphism within *P. cynocephalus anubis* TRIM5α, as there were one synonymous and three nonsynonymous SNPs between the newly characterized sequence and the published sequence [26]. A synonymous variation in nucleotide 90 is polymorphic in both *M. sylvanus* and *P. cynocephalus anubis*.

**Table 2:** Interspecies comparison of predicted TRIM5α amino acid sequence from *M. sylvanus*, *M. mulatta*, and *Papio cynocephalus anubis*.

Amino acid	29	44	69	112	139	142	177	217	247	296	298	310	330	333	339	358	385	423
*Papio*	P	R	Q	S	E	V	D	T	E	V	R	I	T	A	S	I	P	H
*M. sylvanus*	P	K	**R**	**R**	**K**	V/**M**	**E**	**M**	E	A	**C**	**M**	M	**T**	T/**M**	I/**L**	**L**	H/**R**
*M. mulatta*	H	K	Q	S	E	V	D	T	D	A	R	I	M	A/S	T/Ä	I	S	H
Domain	RING			B-Box	Coiled Coil							B30.2/SPRY						

**Bold**  =  not found in P. cynocephalus or M. mulatta.

### TRIMCyp-related sequences evolved once in the Asian macaque lineage

In order to further examine the phylogenetic origin of TRIMCyp-linked sequences, we sequenced *TRIM5* genomic DNA from *P. cynocephalus anubis*, *M. sylvanus*, *M. nigra*, *M. thibetana*, and *M. fascicularis* (GenBank HM468434-HM468446). The sequenced region was amplified using Primers 2 and 4 and consisted of introns 6 and 7 and exons 7 and 8 (see Figure 1). We also analyzed published sequences from *M. mulatta* and *M. nemestrina*. Of the sequences analyzed, four (three *M. fascicularis* and one *M. nemestrina*) had the T allele at the exon 7 splice acceptor, and were known or presumed to be linked to the *CypA* insertion; the remainder had the G allele at the splice site. *M. fascicularis* was the only species for which genomic DNA sequences in this region for both T-containing and G-containing alleles were available. Although some *M. mulatta* have the T allele, a complete sequence for the region analyzed here was not available from publicly accessible data.

**Figure 4:**
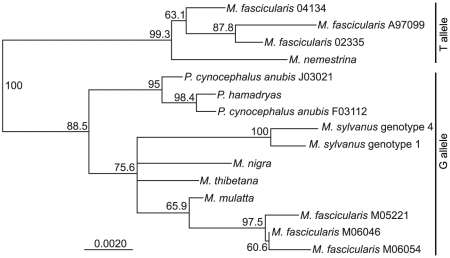
TRIMCyp-linked alleles form a monophyletic group. Phylogenetic tree of *TRIM5* genomic sequences from the exon 6–8 region. Tree was built using a neighbor-joining algorithm using the homologous human sequence as an outgroup (not shown). Bootstrap values from 1000 replicates are shown. Scale bar, substitutions per site.

The four sequences containing the T allele at the exon 7 splice site clearly formed a monophyletic group (Figure 4). In particular, the *M. fascicularis* sequences clustered according to their allele at this site. *M. fascicularis* sequences containing the G allele grouped with *M. mulatta*. Similarly, *M. fascicularis* sequences containing the T allele grouped with *M. nemestrina*. This finding is consistent with the notion that the T allele associated with TRIMCyp expression evolved once, in the common ancestor of *M. fascicularis* and *M. nemestrina* (and thus of all Asian macaques), and has not been subsequently lost in any of the lineages studied.

### TRIMCyp fixation in *M. nemestrina* and *M. leonina* occurred after divergence from *M. nigra*

**Figure 5:**
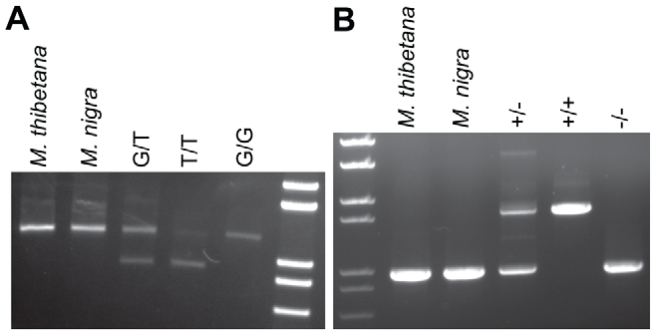
Single *M. thibetana* and *M. nigra* individuals lack TRIMCyp-related genetic changes. (A) *NsiI* restriction site assay on *M. thibetana* and *M. nigra*. (B) PCR across the *TRIM5* 3′ UTR in *M. thibetana* and *M. nigra*. Three *M. fascicularis* of known genotype were used as controls (last 3 lanes of both parts A and B).

To further define TRIMCyp evolution in Asian macaques, we tested one sample each from *M. thibetana* and *M. nigra*. Both of these samples lacked the *CypA* insertion and had the G allele at the exon 7 splice site (Figure 5). Thus, TRIMCyp is absent in at least some individuals of these species. Phylogenetically, *M. thibetana* belongs to the *sinica* group, of which no members have previously been tested. *M. nigra* belongs to the *silenus* group, along with *M. nemestrina* and *M. leonina*. Because the *CypA* insertion and the T allele are fixed in *M. nemestrina* and *M. leonina* [16], [19]–[21], we conclude that TRIMCyp must have become fixed in the *M. nemestrina/M. leonina* lineage after it diverged from *M. nigra*.

## Discussion

**Figure 6:**
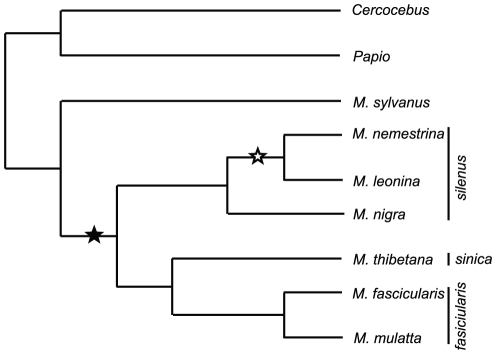
Model for TRIMCyp evolution in Old World primates. Schematic dendrogram showing the history of TRIMCyp evolution inferred here, in the context of established phylogenetic relationships among Old World primate species studied. Filled star, evolution of TRIMCyp. Open star, fixation of TRIMCyp. Asian macaque phylogenetic groups are indicated at right [23]. This graphical representation is not to scale and is not intended to reflect relative divergence. Relationships are adapted from [23], [29], [37].

We report here that TRIMCyp, and the genetic changes required for its expression, are absent in *M. sylvanus* and in two species of baboons. These data, in combination with data on sooty mangabeys [20], suggest that the common ancestor of the macaques also lacked TRIMCyp, and that TRIMCyp evolved after *M. sylvanus* diverged from the Asian macaques (Figure 6). Old World TRIMCyp expression results from two genetic changes that are invariably linked in species examined to date, namely a T allele at the exon 7 splice junction and a *CypA* insertion. The *CypA* insertion required for production of functional TRIMCyp in Old World primates is consistently found in the same genetic location, in the 3′ UTR downstream of exon 8. Furthermore, we show that *CypA*-containing *TRIM5* DNA sequences are monophyletic. Taken together, these data indicate that functional TRIMCyp evolved only once in Old World primates. Given the widespread distribution of TRIMCyp among the Asian macaques and its absence in African primates, we conclude that TRIMCyp expression evolved in the common ancestor of the Asian macaques.

Based on molecular evidence, the macaques are thought to have diverged from the papionin clade about 9–10 million ybp [27], although fossil and geological evidence indicates that this event could have occurred as recently as 6 million ybp [28]. Molecular evidence suggests that the Asian macaques diverged from *M. sylvanus* approximately 5.5–6 million ybp, and diverged from each other about 5–6 million ybp [22], [29]. Based on these data and on our findings, we hypothesize that a retrovirus invaded the population of the Asian macaque progenitors approximately 5–6 million ybp, causing selection for a novel antiretroviral factor and leading to the evolution of TRIMCyp in this clade. This event could have occurred either in Asia or in Europe or Africa, before these species arrived in Asia. The oldest macaque fossil found in Asia is dated at approximately 5.5 million years old, not long after the presumed divergence of Asian from African macaques, suggesting that the migration to Asia was relatively rapid [30]. However, our data do not allow us to pinpoint a location for the evolution of TRIMCyp.

Although our data are most consistent with an origin of TRIMCyp in the common ancestor of Asian macaques, we have also considered several alternative hypotheses. First, it is possible that TRIMCyp was present in ancestral Old World primates but has been lost in all lineages other than Asian macaques. Results shown in Figure 4 clearly show that TRIMCyp sequences have not been lost at the DNA level, by deletion of the *CypA* sequence or by reversion of the exon 7 splice site. If this had occurred in some species, we would expect them to have the G allele but to group with the T-containing sequences, or to have the T allele in the absence of the *CypA* insertion. Neither of these features is present in any of the species tested. Instead, our data show unambiguously that sequences containing the T allele form a monophyletic group, distinct from those containing the G allele. Thus, it is unlikely that TRIMCyp was lost at the DNA level in any lineage.

In contrast, we cannot formally rule out the possibility that TRIMCyp was lost by lineage sorting. In our phylogenetic analysis, the T alleles appear to branch off before the separation of baboon and macaque G alleles (see Figure 4). This could be taken to suggest that the T allele evolved before this evolutionary branching, and thus that TRIMCyp, or at least TRIMCyp-linked sequence changes, may be older than suggested by our other data. However, this analysis is complicated by the possibility of different evolutionary rates in different sequences. In sequences that do not encode TRIMCyp (i.e. those with the G allele), approximately half of the region included in this analysis consists of coding sequence. In sequences containing the T allele, the entire region could be considered to be noncoding and thus potentially under relaxed selection. In these sequences, exon 8 (587 bp) is still used to code for TRIM5η; however, no biological function has been described for this isoform [19]. Due to this uncertainty, no firm conclusions can be drawn from our phylogenetic analysis about the timing of the evolution of the T allele. Thus, the most parsimonious explanation for our data remains that TRIMCyp-related sequences evolved once in the ancestral Asian macaque lineage.

Although it is unlikely that Old World TRIMCyp itself has been lost by lineage sorting, it should be noted that lineage sorting has almost certainly played a part in the evolution of this gene. Expression of functional Old World TRIMCyp requires two genetic changes that must originally have happened independently, namely a single nucleotide transversion and a retrotransposon-mediated insertion. Therefore, ancestral individuals must have had one in the absence of the other. The splice site T allele in the absence of the *CypA* insertion would probably be disadvantageous, since such an animal would be unable to produce either TRIM5α or TRIMCyp, likely making it more susceptible to retroviruses [19]. It is unclear whether the presence of the *CypA* insertion in the absence of the T allele would allow any expression of TRIMCyp. However, under these circumstances, TRIMCyp would likely be only a minor splice variant. Thus, these two genetic changes are expected to confer a strong selective advantage only in combination. The hypothetical ancestral form, with only one of the two genetic changes, has likely been lost by lineage sorting, due either to selection or to genetic drift.

We also considered the possibility that TRIMCyp is present in *M. sylvanus* but was not detected in our study. *M. sylvanus* individuals have been repeatedly introduced into Gibraltar, and animals in this population have mitochondrial haplotypes representative of the most common alleles found in both Algerian and Moroccan populations [24]. All existing wild *M. sylvanus* populations live in these three countries; thus, the Gibraltar population is representative of the species as a whole [24]. Our *M. sylvanus* samples consisted of 36 animals from the Gibraltar colony. This colony currently contains approximately 230 animals belonging to six groups, with group sizes ranging from 14–64 individuals per group. We sampled individuals from all six groups. The animals in our sample also have diverse *TRIM5* sequences, so they do not represent closely related animals with similar or identical genotypes. Statistically, the absence of TRIMCyp in any of our 36 sample animals implies that the prevalence of TRIMCyp in our population of 230 is no higher than 8.3 percent or 19 animals (p<0.05, according to the binomial probability distribution). Thus, although we cannot rule out the possibility that TRIMCyp is present in less than 10% of the Gibraltar population, or that it is a rare genotype in African *M. sylvanus* that is not present in the Gibraltar population, we believe that the available data are best explained by a model in which TRIMCyp evolved after the divergence of *M. sylvanus* from the Asian macaques.

A final alternative hypothesis is that TRIMCyp evolved in one group of Asian macaques after their divergence from other Asian macaques, and entered other groups by hybridization and introgression. A scenario in which TRIMCyp evolved specifically in the *silenus* group, which includes *M. nemestrina* and *M. leonina*, and later entered the *fascicularis* group, could explain the fact that it is fixed in *M. nemestrina* and *M. leonina* but not in *M. mulatta* or *M. fascicularis*. However, we believe that this hypothesis is not plausible. Although there is extensive literature on introgression between *M. fascicularis* and *M. mulatta*, there is currently no evidence of introgression between more distant macaque groups [29], [31]. Further, the geographic range of *M. nemestrina* and *M. leonina* overlaps with that of Chinese, but not Indian, *M. mulatta*. This contrasts with the presence of TRIMCyp in Indian but not Chinese *M. mulatta*. Thus, we believe that the most plausible scenario is that TRIMCyp evolved in the common ancestor of Asian macaques, and is not present in *M. sylvanus* or other Old World monkeys. We suggest that its variable frequency in different taxa results from the complex selective pressures exerted by multiple and different retroviral challenges.

Although TRIMCyp expression likely conferred a selective advantage on Asian macaque ancestors, it did not become fixed in the general Asian macaque population. TRIMCyp expression is polymorphic in both *M. fascicularis* and *M. mulatta*, and absent in individuals of *M. thibetana* and *M. nigra* reported here. TRIM5α alleles are thought to be subject to balancing selection in Old World monkeys, based on the existence of ancient shared polymorphisms [7], and it seems likely that TRIMCyp is subject to similar evolutionary pressures. Thus, if animals are subject to challenge both by retroviruses that are susceptible to TRIM5α and by those susceptible to TRIMCyp, the maintenance of both restriction factors in the population would be beneficial. Our dataset does not provide molecular evidence to support the activity of balancing selection by Tajima's *D* test [32] (data not shown); however, the dataset is small, and its power to detect such selection is low. Thus, it is possible that the long-term maintenance in some Asian macaque species of both TRIM5α and TRIMCyp-expressing alleles may be due to balancing selection. Alternatively, there may be direct advantages to heterozygosity in this retroviral restriction factor, which could have led to the maintenance of both alleles.

*M. sylvanus* lacks many common viruses enzootic to other macaque species, including herpesviruses (cytomegalovirus and Cercopithecine herpesvirus) as well as retroviruses (simian immunodeficiency virus, simian retrovirus, and simian T cell leukemia virus) [33]. The only retrovirus known to exist in this species is simian foamy virus [33]. Because *M. sylvanus* does not normally have contact with other nonhuman primate species, its lack of retroviruses commonly found in Asian macaques may simply be due to a lack of exposure. However, we might also speculate that *M. sylvanus* has in fact evolved resistance to some or all of them. The many species-specific polymorphisms in *M. sylvanus* TRIM5α, some of which are in regions known to be important for antiviral specificity, could be a result of such evolution. However, the species-specific SNPs in *M. sylvanus* TRIM5α could also represent a change in specificity or a loss of antiviral activity through genetic drift. Functional studies will help to elucidate these possibilities.

TRIMCyp in New World primates evolved independently from its counterpart in Old World primates. Based on its presence in all members of the genus *Aotus* and its absence from others species, New World TRIMCyp must have evolved between 4.5 and 22 million ybp [34]. This date range encompasses the 5–6 million ybp proposed here for the evolution of Old World TRIMCyp. Available data do not allow us to distinguish whether the concurrent evolution of TRIMCyp in these two lineages was due to a worldwide retroviral epidemic or to multiple and separate events. However, findings reported here, together with existing evidence [22], [23], [30], allow us to define with unprecedented precision the time and possible geographic origin for the evolution of TRIMCyp in Old World primates. These data also provide evidence linking the evolution of an antiretroviral restriction factor with a speciation event, namely the divergence of Asian macaques from the *M. sylvanus* lineage. Understanding the evolution of host restriction factors among macaque species will elucidate natural antiviral mechanisms and help us to better use these species as animal models for retroviral diseases such as HIV/AIDS.

## Materials and Methods

### Ethics Statement

All animal-related work has been conducted according to the Public Health Services Policy on Humane Care and Use of Laboratory Animals (http://grants.nih.gov/grants/olaw/references/PHSPolicyLabAnimals.pdf). Washington National Primate Research Center (WaNPRC) is an AAALAC-accredited institution. All experimental protocols were reviewed and approved by the University of Washington's Institutional Animal Care and Use Committee (4233-01 and 4202-03) and by the Gibraltar Ornithological and Natural History Society. Peripheral blood was collected by venipuncture when animals were under sedation to relieve pain and suffering. Biological samples were collected and transported according to all relevant national and international guidelines.

### Samples

DNA samples were obtained from the Coriell Institute Integrated Primate Biomaterials and Information Resource (IPBIR) for *M. nigra* (Repository number PR00726), *M. thibetana* (PR00711), and *P. hamadryas* (PR00559). Peripheral blood (maximum of 10 ml/kg/week) from *P. cynocephalus anubis* and *M. fascicularis* animals housed at the WaNPRC was collected in heparinized Vacutainer tubes. *M. sylvanus* were captured from wild populations in Gibraltar, and blood was collected in EDTA-coated Vacutainer tubes. Blood was collected by venipuncture when animals were under sedation (ketamine 10–15 mg/kg) to relieve pain and suffering. DNA was isolated using a QIAamp DNA Blood Mini kit (Qiagen) on fresh whole blood. RNA was isolated using a QIAamp RNA Blood Mini kit (Qiagen) on fresh whole blood (*P. cynocephalus anubis* and *M. fascicularis*) or frozen leukocyte pellets preserved using RNAlater (Ambion) (*M. sylvanus*).

### PCR and Restriction assay

For the *NsiI* restriction assay, genomic DNA was amplified using Platinum PCR Supermix High Fidelity (Invitrogen), with forward primer MfaT5ex6F (Primer 2; ATC TGA AAC GAA TGC TAG ACA TG) and reverse primer 3TrmNotI (Primer 4) (ATC TAG GCG GCC GCT TAA GAG CTT GGT GAG CAC AGA GTC ATG). PCR products were digested using FastDigest *Nsi*I enzyme (Fermentas). All products were run on a 1.2% agarose gel and visualized using ethidium bromide.

To test for the *CypA* insertion, genomic DNA was PCR amplified using Platinum PCR Supermix High Fidelity (Invitrogen), with forward primer 3UTRF (Primer 3; TGA CTC TGT GCT CAC CAA GCT CTT G) and reverse primer 3UTRRLong (Primer 6; TCA CCC TAC TAT GCA ATA AAA CAT TAG GAC), as described by Wilson *et al.* [18]. PCR products were run on a 1% agarose gel.

For RT-PCR, first-strand cDNA was produced using the Accuscript High Fidelity 1^st^ Strand cDNA Synthesis Kit (Stratagene), using random hexamers as primers. The cDNA was then PCR amplified using AccuPrime Pfx Supermix (Invitrogen), with forward primer XhoITRIM5 (Primer 1; CTA GAT CTC GAG ATG GCT TCT GGA ATC CTG GTT AAT GTA AAG) and reverse primers 3TrmNotI (Primer 4; for TRIM5) or CypARMCSNotI (Primer 5; GTA TAT GCG GCC GCT TAT TCG AGT TGT CCA CAG TCA G) (for TRIMCyp).

### Cloning and Sequencing

PCR products were cloned using a StrataClone Blunt PCR Cloning Kit (Stratagene). Sequencing was performed by the University of Washington Pharmaceutics Sequencing Center.

### Sequence Analysis

Sequence analysis was performed using Geneious 4.8 [35] and MEGA 4.0 software [36]. Sequences were aligned for a 1204 bp genomic region of TRIM5α encompassing exons 7 and 8 and introns 6 and 7 (amplified using primers 2 and 4). The sequences were derived from nine *M. fascicularis*, one *M. nigra*, one *M. thibetana*, one *P. hamadryas*, and two *P. cynocephalus anubis* as well as from one *M. mulatta* (extracted from chromosome 14 of the rhesus macaque genome, NC_007871.1), one *M. nemestrina* (EU371641.1), and the homologous *H. sapiens* sequence (extracted from chromosome 11 of build 37.1 of the human genome, NT_009327.18) obtained from GenBank. A phylogenetic tree of this region was generated in Geneious using a neighbor-joining algorithm with 1000 bootstrap replicates. The human sequence was used as an outgroup.

MEGA4.0 software was used to detect departures from neutrality with Tajima's *D* statistic [32]. Positions containing gaps and missing data were deleted.

Sequences generated in this work were deposited in GenBank (Accession numbers HM468429- HM468446).
